# *IRAK4* and *TLR3* Sequence Variants may Alter Breast Cancer Risk among African-American Women

**DOI:** 10.3389/fimmu.2013.00338

**Published:** 2013-10-29

**Authors:** Susan T. Yeyeodu, LaCreis R. Kidd, Gabriela M. Oprea-Ilies, Brian G. Burns, Tiva T. VanCleave, Joong-Youn Shim, K. Sean Kimbro

**Affiliations:** ^1^Department of Biology, Julius L. Chambers Biomedical/Biotechnology Research Institute, North Carolina Central University, Durham, NC, USA; ^2^Department of Pharmacology and Toxicology, University of Louisville, Louisville, KY, USA; ^3^Winship Cancer Institute, Emory School of Medicine, Emory University, Atlanta, GA, USA

**Keywords:** breast cancer, innate immunity, single nucleotide polymorphism, TLR3, IRAK4, African-American, XCT subdomain, extracellular kinase

## Abstract

Mounting evidence suggests that imbalances in immune regulation contribute to cell transformation. Women of African descent are an understudied group at high risk for developing aggressive breast cancer (BrCa). Therefore, we examined the role of 16 innate immune single nucleotide polymorphisms (SNPs) in relation to BrCa susceptibility among 174 African-American women in Atlanta, GA, USA. SNPs were examined in germ-line DNA collected from 102 BrCa patients and 72 women with benign nodules using SNPstream methodology. Inheritance of the *TLR3* rs10025405 GG genotype was associated with an 82% decrease in BrCa risk. In contrast, individuals who possessed at least one *IRAK4* rs4251545 T allele had a 1.68- to 4.99-fold increase in the risk of developing BrCa relative to those with the referent genotype (OR = 4.99; 95% CI = 1.00, 25.00; *p* = 0.0605). However, the *IRAK4* rs4251545 locus was only significant under the additive genetic model (*p* trend = 0.0406). *In silico* predictions suggest *IRAK4* rs4251545 SNP falls within a transcription enhancer/silencer region of the gene and codes for an Ala428Thr amino acid change. This missense mutation introduces a potential phosphorylation site in the extreme carboxy terminus (XCT) of the IRAK4 kinase domain. Preliminary molecular modeling predicts that this SNP stabilizes two alpha helices within the XCT on the surface of the IRAK4 kinase domain and increases the size of the groove between them. Our *in silico* results, combined with previous reports noting the presence of IRAK4 and XCT fragments in mouse and human serum, suggest the possibility that the XCT subdomain of IRAK4 possesses biological function. These findings require further evaluation and validation in larger populations, additional molecular modeling as well as functional studies to explore the role of IRAK4 and its XCT in cell transformation and innate immunity.

## Introduction

Breast cancer (BrCa) is the most common cancer morbidity among women worldwide and is especially prevalent in the US and other developed countries. Several factors contribute to BrCa risk, including age, family history of BrCa, personal history of benign breast disease, late menopause, obesity, high endogenous estrogen and testosterone levels, adult weight gain, early menarche and null parity, and *BRCA 1/2* mutations. Specific mutations in *BRCA1* and *BRCA2* are associated with a genetic predisposition to BrCa. However, these mutations account for only 5–10% of the reported BrCa cases (1). Exploring the contribution of other genetic factors associated with BrCa is an active area of research (2). In fact, compelling evidence has emerged that suggests genetic anomalies in inflammatory and immune response pathways may lead to cellular transformation and contribute to BrCa pathogenesis (3, 4).

**Figure 1:**
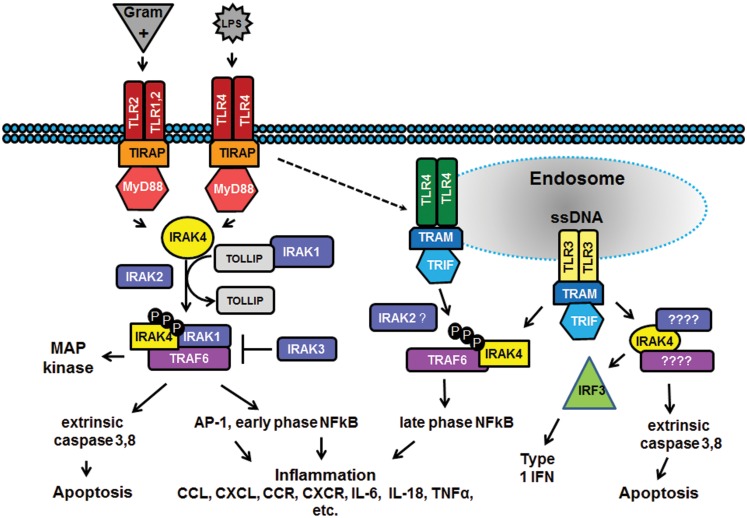
**Signal transduction pathways involving TLR3 and IRAK4**. Surface membrane Toll-like receptors (TLRs, in red) such as TLR1, 2, and 4 respond to specific extracellular pathogen-associated molecular patterns (PAMPs, in gray), including Gram-positive bacteria (recognized by TLR2 homo- or hetero-dimers with TLR1/TLR6/TLR10) and lipopolysaccharide (LPS, recognized by TLR4 homo- or hetero-dimers with TLR2 or TLR1/TLR6/TLR10) to initiate inflammatory and/or apoptotic signaling pathways. Endosomal TLRs such as TLR4 (green) and TLR3 (yellow) bind pathogenic fragments, initiating pro-inflammatory responses, extrinsic caspase-induced apoptosis, and/or expression of interferon (IFN) regulatory transcription factors [e.g., interferon regulatory factor 3 (IRF3), light green] and production of type I interferon. Interleukin-1 receptor-associated kinase 4 (IRAK4, in yellow) mediates signaling in TLR pathways by responding to myeloid differentiation primary response gene 88 (MyD88, dark orange) dependent signals on the cell surface and Toll-interleukin-1 receptor (TIR)-domain-containing adapter-inducing interferon-β (TRIF, in light blue) dependent signals in endosomes. Engagement of IRAK4 with these sorting receptors stimulates autophosphorylation (black “P”) followed by phosphorylation of IRAK1 and/or IRAK2, tumor necrosis factor receptor-associated factor 6 (TRAF6) activation and a wide variety of downstream regulatory pathways via such message integrators as nuclear factor kappa beta (NFkB) and mitogen-activated protein (MAP) kinases. TLR3 (in yellow) recognition of dsRNA (SS) initiates both TRAF6-dependent (in purple) stimulation of NFkB to generate a pro-inflammatory response and TRAF6–independent stimulation of interferon (IFN) production and extrinsic caspase-driven apoptosis; IRAK4 participates in both TRAF6-dependent and -independent TLR3 responses.

Innate immune capabilities enable most tissues, including breast epithelia, to mount a first response to infection through several surface and intracellular membrane receptors (5). Best characterized among these are the Toll-like receptors (TLRs) that recognize pathogens, dietary or environmental toxins, or endogenous biomolecules that possess specific pathogen-associated molecular patterns (PAMPs) or damage-associated molecular patterns (DAMPs). TLRs are triggered as homo- or hetero-dimers and complex with adaptor molecules that recruit regulatory kinases to initiate downstream cellular responses, as summarized in Figure 1. Among these regulatory kinases, IL-1 receptor-associated kinase 4 (IRAK4) is widely employed, and transduces signals from TLRs 1 through 10.

Recent evidence links TLRs and their signaling pathways with tumor progression (6–9). Enhanced TLR expression at the cell surface and in endosomes has been observed in several human cancers, including BrCa (10, 11). Total and surface expression of TLR2 are both enhanced in the highly metastatic MDA-MB-231 human BrCa cell line relative to the less aggressive MDA-MB-468 or untransformed MCF10A cell lines (12). Further, bacterial peptidoglycan stimulation of TLR2 promotes BrCa invasiveness (12). Consistent with these results, TLR4 knockdown in MDA-MB-231 cells inhibits cell proliferation and inflammatory cytokine secretion (13). Conversely, treatment of MCF7 human BrCa cells with agonists to TLR3 and TLR7 (both located in intracellular endosomes) inhibits BrCa cell growth that is reversible by treatment with the autophagy inhibitor 3-methyladenine (14). Similarly, activation of TLR5 by flagellin inhibits the growth of MCF7 and MDA-MB-468 BrCa cell lines (15) and activation of endosomal TLR9 with agonist oligo(CpG) inhibits estrogen-induced growth of MCF7 cells (16). While the precise mechanisms are poorly understood, these findings suggest that anomalies in TLR signaling pathways influence BrCa progression.

Consistent with this, TLR-related sequence variants have been implicated in BrCa susceptibility. Only a few population-based studies have detected single nucleotide polymorphisms (SNPs) in genes related to TLR signaling that alter BrCa risk. Among Korean women, a modest increase in BrCa risk (OR = 1.63; 95% CI = 1.14, 2.34) was linked with possession of one or more of the *IRAK3* rs1732877 minor alleles (17). Similarly, presence of the minor variant of the non-synonymous *TLR4* rs4986790 A/G SNP, in which aspartic acid 299 is replaced by glycine, was associated with a modest increase in BrCa risk (OR = 1.81; 95% CI = 1.23, 2.66) in a Greek cohort (18). Finally, inheritance of the homozygous minor variant of the *TLR1/TLR6/TLR10* rs7696175 locus (situated in proximity to all three *TLR* promoters) was associated with a large fourfold increase in BrCa risk (OR = 4.11; 95% CI = 1.28, 13.24) among African-American women as opposed to little or no risk among women of European ancestry (OR = 1.20; 95% CI = 0.93, 1.53) (19). Only the last of these studies addressed BrCa risk among women of African descent, despite the fact that this sub-group suffers disproportionately from more aggressive forms of BrCa (20, 21) and is underrepresented in BrCa research efforts (22).

The study of the biological/genetic factors that contribute to disparities in BrCa risk and severity among women of various races is a nascent science, and to date has focused most heavily on differences in hormone receptor expression (2). Barnholtz-Sloan et al. (19) explored a large panel of SNPs from genes with a variety of functions that had been previously reported as BrCa susceptibility genes in genome wide association (GWAS) studies (19). Notably, the minor allele frequency of the *TLR1/TLR6/TLR10* rs7696175 allele in the Barnholtz-Sloan study is highly disparate between women of African and European descent (3 and 44%, respectively). Consequently, we selected additional TLR-pathway associated SNPs, in part, based on significant disparities in minor allele frequency (≥10% difference) between individuals of African and European descent (Table 1). In the current study, we evaluated 16 TLR-associated SNPs in relation to BrCa among 174 African-American female participants (102 BrCa cases, 72 benign controls).

**Table 1:** **Prevalence of selected innate immunity sequence variants and their functional consequence**.

dbSNP ID	Gene	NT change	MAF AA (%)	Major/major genotype *n* (%) AA	Major/minor genotype *n* (%) AA	Minor/minor genotype *n* (%) AA	MAF Cau (%)	Major/major genotype *n* (%) Cau	Major/minor genotype *n* (%) Cau	Minor/minor genotype *n* (%) Cau	*P*-value^a^ AA vs. Cau
rs10025405	TLR3	A > G	G = 31.6	24 (49.0)	19 (38.8)	6 (12.2)	G = 42.0	39 (34.5)	53 (46.9)	21 (18.6)	0.205
rs10759930	TLR4	C > T	T = 8.7	19 (82.6)	4 (17.4)	0 (0.0)	T = 41.7	7 (29.2)	14 (58.3)	3 (12.5)	0.0006
rs11672931	TICAM1 (aka TRIF)		G = 23.8	12 (57.1)	8 (38.1)	1 (4.8)	G = 27.3	13 (59.1)	6 (27.3)	3 (13.6)	0.606
rs242724	IRAK2	A > C	C = 22.7	6 (54.5)	5 (45.5)	0 (0.0)	C = 31.0	56 (49.6)	44 (38.9)	13 (11.5)	0.735
rs2569188	CD14	A > G	G = 39.8; A = 60.2	18 (36.7)	23 (46.9)	8 (16.3)	A = 49.1	24 (21.4)	62 (55.4)	26 (23.2)	0.118
rs4251545	IRAK4	G > A	A = 32.6	11 (47.8)	9 (39.1)	3 (13.0)	A = 10.4	19 (79.2)	5 (20.8)	0 (0.0)	0.036
rs4684672	IRAK2	G > A	A = 22.7	14 (63.6)	6 (27.3)	2 (9.1)	A = 39.5	7 (36.8)	9 (47.4)	3 (15.8)	0.224
rs4696480	TLR2	T > A	A = 39.6	8 (33.3)	13 (54.2)	3 (12.5)	A = 46.8	8 (25.8)	17 (54.8)	6 (19.4)	0.759
rs4833095	TLR1	C > T	T = 34.8; C = 65.2	11 (47.8)	8 (34.8)	4 (17.4)	C = 20.8	15 (62.5)	8 (33.3)	9 (4.2)	0.577
rs4986790	TLR4	A > G	G = 3.3	20 (83.3)	4 (16.7)	0 (0.0)	G = 4.4	29 (93.5)	2 (6.5)	0 (0.0)	0.387
rs5743899	TOLLIP	A > G	G = 34.8	9 (39.1)	12 (52.2)	2 (8.7)	G = 17.9	75 (67.0)	34 (30.4)	3 (2.7)	0.019
rs6442161	IRAK2	C > T	T = 21.9	30 (62.5)	15 (31.2)	3 (6.2)	T = 42.5	41 (36.3)	48 (42.5)	24 (21.2)	0.00400
rs7251	IRF3	C > G	G = 25.0; C = 75.0	14 (58.3)	8 (33.3)	2 (8.4)	C = 37.1	21 (36.2)	31 (53.4)	6 (10.3)	0.205
rs7045953	TLR4	A > G	G = 17.4	15 (65.2)	8 (34.8)	0 (0.0)	G = 14.6	80 (70.8)	33 (29.2)	0 (0.0)	0.623
rs7657186	TLR3	G > A	A = 25.0	13 (54.2)	10 (41.7)	1 (4.2)	A = 24.5	18 (60.0)	9 (30.0)	3 (10.0)	0.578
rs913930	TLR4	A > G	G = 28.3	14 (60.9)	5 (21.7)	4 (17.4)	G = 38.1	48 (42.5)	44 (38.9)	21 (18.6)	0.198

MAF, minor allele frequency; AA, African-American/African Ancestry; Cau, Caucasian;

*^a^ The Fisher’s Exact test or chi-square test of homogeneity was used to compare the genotype frequencies from women of African and European descent using data from NCBI*.

## Materials and Methods

### Study population and tumor characterization

Study participants, including 102 BrCa cases and 72 benign controls were enrolled from Grady Memorial Hospital and Emory Midtown Hospital in Atlanta, GA, USA. All participants self-identified as African-Americans and provided written informed consent for participation in genetic analysis studies under protocol #20417 approved by Emory University’s Institutional Review Board. Socioeconomic status, nutrition, environmental exposure, and other confounding factors were not addressed. Tumor size was measured and tumor stage and invasiveness/morphology were determined according to standard histopathological definitions. Estrogen (ER) and progesterone receptor (PR) and Her-2 neu expression were determined by immunohistochemistry staining (23).

### Criteria for TLR signaling gene and SNP selection

**Figure 2:**
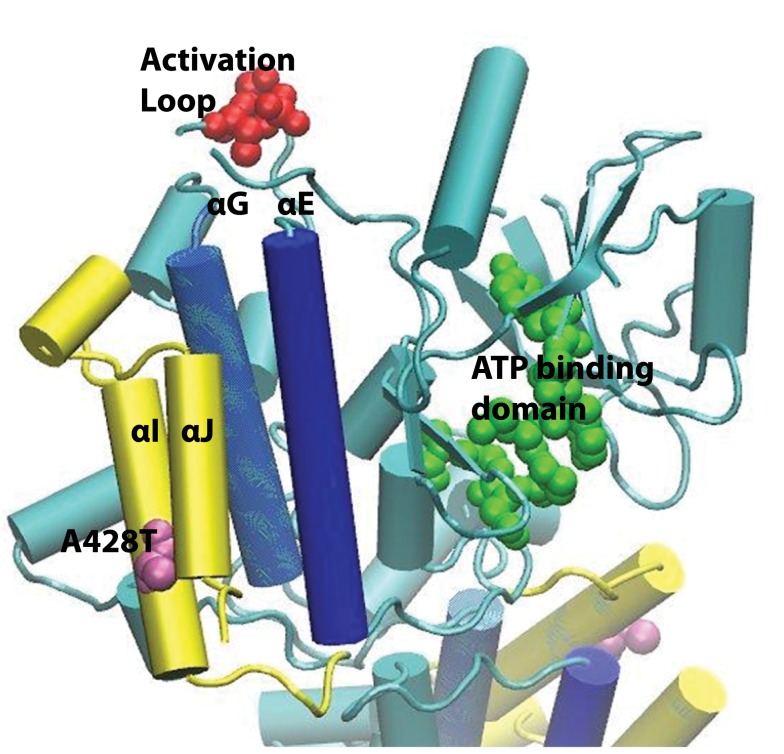
**Structural features of the IRAK4 kinase domain**. The Ala428Thr (mauve) non-synonymous SNP is located on αI, the penultimate alpha helix, facing the carboxy terminal αJ as part of the putative extreme carboxy terminal (XCT) subdomain (yellow). Preliminary molecular dynamics simulations superpositioned backbone atoms of αI (yellow), αG (blue), and αE (blue) to evaluate local structural changes introduced by replacing Ala428 with Thr. The location of key residues in known functional domains, including Thr342, Thr345, and S346 of the activation loop (red) and amino acids 262 through 269 in the ATP binding domain (green) are also indicated.

Toll-like receptor-associated genes and SNPs were selected using one or more of the following criteria: (1) epidemiological or molecular biological evidence from published reports indicating a relationship between the SNP/gene with cancer or inflammatory/immune response related diseases (Figure 1); (2) commonly studied loci; (3) marked disparities in genotype frequency comparing women of African descent to their Caucasian counterparts (i.e., ±10% change) (Table 1); (4) evidence demonstrating a link with alterations in mRNA expression/stability or protein expression/structure or function using *in silico* tools or published reports^1^; and (5) a minor allele frequency ≥1% reported in the National Center for Biotechnology Information (NCBI) Entrez SNP (24)^2^. The current study focused on 16 SNPs detected in 10 TLR genes as well as downstream markers associated with TLR mediated signaling pathways (i.e., *TLR1-4, CD14, IRAK2&4, IRF3, TICAM1, TOLLIP)*, as described in Table 1.

### SNPstream genotyping

Sixteen candidate SNPs among African-American subjects were genotyped using the GenomeLab™ SNPstream^®^ Genotyping System (Beckman Coulter, Brea, CA, USA) within the Center for Medical Genomics at Emory University. SNPstream executes high-throughput multiplex genotyping using single-base fluorescent primer extension. Primers were designed using autoprimer tools^3^. Following primer design, allelic discrimination was facilitated, in part, by PCR amplification of 100 base pairs flanking each SNP using 384-well PCR plates. GenomeLabSNPstream Genotyping System Software Suite v2.3 was used for image processing and genotype base calling.

### Statistical analysis

Logistic regression (LR) analysis was used to evaluate innate immunity associated SNPs among African-Americans in relation to BrCa risk. To assess whether individuals possessing innate immune sequence variants influence the risk of developing BrCa, we tested for significant differences in the distribution of homozygous major, heterozygous, or homozygous minor genotypes between cases and controls using the chi-square test of homogeneity. The associations between BrCa risk and selected polymorphic genes, expressed as odds ratios (ORs) and corresponding 95% confidence intervals (CIs), were estimated using unconditional multivariate LR models. Risk estimates were adjusted for age. LR analyses for genetic variants and BrCa development were conducted using the major/common genotype as the reference group. All chi-square and LR analyses were conducted using SAS 9.1.3 (SAS Institute, Cary, NC, USA). Statistical significance required a *P*-value <0.05.

### Molecular modeling

Molecular dynamics (MD) simulations were performed to examine the effect of the *IRAK4* rs4251545 Ala428Thr variant on enzyme structure, as described previously (25), as described previously. Briefly, simulations were performed with NAMD parallel, object-oriented high-performance software (ver. 2.6b2 for Linux-Power-MPI). CHARMM22 force field parameters were used for the protein along with the three-site TIP3 rigid water model. Initial coordinates of IRAK4 were obtained from the x-ray structure of the human IRAK4 kinase domain from amino acids 160–460 (PDB code: 2OIB) (26). A model of the *IRAK4* rs4251545 variant was generated by replacing Ala428 in IRAK4 with a Thr residue. After immersion of IRAK4 in a rectangular cell of water molecules to produce a periodic box of dimension 93 Å × 89 Å × 96 Å, the variant enzyme system was subjected to 2,500 steps of minimization. The minimized structure was then simulated for 5 ns in a constant temperature and pressure ensemble. The temperature was maintained at 310°K through the use of Langevin dynamics with a damping coefficient of 1/ps. The pressure was maintained at one ATM by using the Nosé–Hoover method. The van der Waals interactions were switched at 10 Å and zeroed smoothly at 12 Å. Electrostatic interactions were monitored using the Particle Mesh Ewald (PME) method (27). A pair list for calculating the van der Waals and electrostatic interactions was set to 13.5 Å and updated every ten steps. The timestep size for integration of each step of the simulation was 1 fs. The resulting variant was compared with the wild-type enzyme structure.

## Results

### Characterization of patients and their tumors

The patient and tumor characteristics of our study participants are summarized in Table 2. For this pilot study based in Atlanta, GA, we enrolled 174 African-American women who had been diagnosed with BrCa or who possessed benign nodules (control). At enrollment, BrCa patients were approximately 10 years older than women with benign disease (<0.0001); about 66% of the cases were diagnosed after age 50. The majority of the breast tumors were characteristic of earlier stages (Stage 0–II) of disease (91.8%) with invasive pathologies (64.7%), namely invasive lobular or uninvasive ductal carcinomas. Further, more than half of the cases lacked expression of estrogen receptor (31.6%), PR (39.8%), or Her-2 neu (87.3%), resulting in 29.6 and 22.1% double or triple negative breast tumors, respectively.

**Table 2:** **Patient and tumor characteristics**.

Characteristics	Cases *n* (%)	Controls *n* (%)	*p*-Value
Median age at enrollment (range)	57 (24–89)	47 (18–78)	<0.0001
Age at enrollment (years)
>70	20 (19.6)	6 (8.3)	0.0002
61–70	17 (16.7)	5 (6.9)	
51–60	35 (34.3)	20 (27.8)	
41–50	23 (22.5)	19 (26.4)	
≤40	7 (6.9)	22 (30.6)	
Age at diagnosis (years)
>70	7 (6.9)		
61–70	23 (22.5)		
51–60	37 (36.3)		
41–50	16 (15.7)		
≤40	19 (18.6)		
Tumor stage
0	35 (35.7)		
I	30 (30.6)		
II	25 (25.5)		
III	3 (3.1)		
IV	5 (5.1)		
Not determined	4 (3.9)		
Tumor size (cm)
<2	53 (60.9)		
≥2	34 (39.1)		
Not determined	15 (14.7)		
Nodal status
Negative	45 (68.2)		
Positive	21 (31.8)		
Not determined	36 (35.3)		
Pathology
Non-invasive (DCIS, LCIS)	36 (35.3)		
Invasive (IDC, ILC)	66 (64.7)		
Not determined	0 (0.0)		
Estrogen receptor (ER)
Positive	67 (68.4)		
Negative	31 (31.6)		
Not determined	4 (3.9)		
Progesterone receptor (PR)
Positive	59 (60.2)		
Negative	39 (39.8)		
Not determined	4 (3.9)		
Her-2 neu
Positive	9 (12.7)		
Negative	62 (87.3)		
Not determined	31 (30.4)		
Triple negative BrCa^a^
No	74 (77.9)		
Yes	21 (22.1)		
Not determined	7 (6.9)		
Double negative BrCa^b^
No	69 (70.4)		
Yes	29 (29.6)		
Not determined	4 (3.9)		
Surgery status
No surgery(biopsy only)	3 (2.9)		
Lumpectomy	33 (32.4)		
Partial mastectomy	25 (24.5)		
Total mastectomy	34 (33.3)		
Modified radical mastectomy	6 (5.9)		
Unknown	1 (1.0)		

^a^ ER-, PR-, Her2-

^b^ ER-, PR-

### Prevalence of minor alleles/genotype frequency

The prevalence of the 16 *TLR* variants was fairly common among the women of African descent in our study, based on a minor allele frequency ≥5%. With the exception of the functional Ala299Gly *TLR4* rs4986790 variant, the minor allele frequencies among controls in our study set ranged between 8.7 and 39.8%, with a median of 22.0%. Approximately 16% of the women possessed one copy of the *TLR4* rs4986790 A/G genotype, with a minor allele frequency of 3.3%. For 15 out of 16 innate immunity SNPs, there were no significant departures in the observed genotype frequencies among controls when compared with expected frequencies under the Hardy–Weinberg Equilibrium (HWE) [*p* ≥ 0.4833]. Moreover, the prevalence of the genotypes among controls was comparable to those reported for women of African descent in NCBI SNP Entrez (*p* ≥ 0.056) (24). Only the *TICAM1* rs11672931 variant deviated from HWE (*p* = 0.0027), despite the fact that its allele (27.3%) and genotype frequency among our study participants corroborated with NCBI values (24).

### Relationship between TLR-associated sequence variants and breast cancer risk

SNPstream analysis of 16 innate immunity SNPs among African-American women with BrCa (*n* = 102) and benign disease (*n* = 72) identified 4 loci whose genotype frequencies were altered with respect to BrCa risk (Table 3). In the unadjusted models, three of these were associated with a decrease in BrCa risk. Possession of one or more minor *TLR2* rs4696480 A, *TLR3* rs10025403 G, and *IRAK2* rs6442161 T alleles was associated with a 45–79% reduction in the risk of developing BrCa. Among these three loci, statistical significance was observed only for the TLR3 rs10025403 marker under the unadjusted and/or age-adjusted dominant (OR = 0.18, 95% CI = 0.05, 0.70); *p* = 0.0123), recessive (OR = 0.20, 95% CI = 0.06, 0.75; *p*-value = 0.0143), and additive (unadjusted *p*-value for trend = 0.0337) genetic models.

**Table 3:** **Innate immune SNPs associated with BrCa risk among African-American women**.

Genes	dbSNP ID location predicted function	Genotype	Cases *n* (%)	Controls *n* (%)	NCBI AA	OR (95% CI)	Adj OR (95% CI)	*p*-Value	*p*-Value for trend
*TLR2*	rs4696480	TT	47 (46.1)	23 (31.9)	0.33	1.00 (reference)	1.00 (reference)	0.1357	0.1959
	Intron 1	TA	37 (36.3)	36 (50.0)	0.54	0.50 (0.26, 0.99)	0.56 (0.27, 1.18)	0.0469	
	1685bp from 5’	AA	18 (17.6)	13 (18.1)	0.13	0.68 (0.28, 1.62)	0.53 (0.21, 1.34)	0.3808	
		≥1 A allele	55 (53.9)	49 (68.1)	0.67	0.55 (0.29, 1.03)	0.55 (0.28, 1.09)	0.0624	
		AA vs (TT + TA)				0.97 (0.44, 2.14)	0.70 (0.30, 1.65)	0.9446	
*TLR3*	rs10025405	AA	54 (53.0)	31 (43.1)	0.66	1.00 (reference)	1.00 (reference)	0.0276	0.0337
	3’ near gene	AG	44 (43.1)	30 (41.6)	0.31	0.84 (0.44, 1.59)	0.80 (0.40, 1.62)	0.5987	
		GG	4 (3.9)	11 (15.3)	0.04	0.21 (0.06, 0.71)	0.18 (0.05, 0.70)	0.0123	
		≥1 G allele	48 (47.0)	41 (56.9)	0.35	0.67 (0.37, 1.23)	0.64 (0.33, 1.24)	0.1997	
		GG vs (AA + AG)				0.23 (0.07, 0.74)	0.20 (0.06, 0.75)	0.0143	
*IRAK2*	rs6442161	CC	56 (54.9)	33 (45.8)		1.00 (reference)	1.00 (reference)	0.1058	0.0721
	Intron 1	CT	40 (39.2)	28 (38.9)		0.84 (0.44, 1.61)	1.05 (0.52, 2.13)	0.6018	
		TT	6 (5.9)	11 (15.3)		0.32 (0.11, 0.95)	0.32 (0.10, 1.11)	0.0401	
		>1 T allele	46 (45.1)	39 (54.2)		0.70 (0.38, 1.27)	0.85 (0.44, 1.64)	0.2393	
		TT vs (CC + CT)				0.35 (0.12, 0.99)	0.32 (0.10, 1.05)	0.0469	
*IRAK4*	rs4251545	CC	50 (49.0)	44 (62.0)	0.46	1.00 (reference)	1.00 (reference)	0.0991	0.0406
	Exon	CT	42 (41.2)	25 (35.2)	0.39	1.51 (0.80, 2.86)	1.68 (0.83, 3.40)	0.2042	
	Splicing	TT	10 (9.8)	2 (2.8)	0.15	4.50 (0.94, 21.6)	4.99 (1.00, 25.00)	0.0605	
	(ESE, ESS)	≥1 T allele	52 (51.0)	27 (38.0)	0.54	1.70 (0.92, 3.14)	1.90 (0.97, 3.75)	0.0936	
	Ala428Thr	TT vs (CT + TT)				3.75 (0.80, 17.66)	3.96 (0.81, 19.3)	0.0947	

*AA, African-American/African ancestry; OR, unadjusted odds ratio; Adj OR, adjusted odds ratio; CI, confidence interval*.

On the other hand, inheritance of two copies of the *IRAK4* rs4251545 TT genotype resulted in a fivefold increase in BrCa susceptibility relative to those with the referent genotype (OR = 4.99; 95% CI = 1.00, 25.00; *p* = 0.0605); however, the findings were not statistically significant. Notably, under the additive genetic model, we observed a significant “dose-response” between the number of alleles in the genetic model (i.e., 0, 1, 2) and the risk estimates (*p*-value for trend = 0.0406) for this SNP.

### Functional consequences of *TLR3* rs10025405 and *IRAK4* rs4251545

Inheritance of the homozygous minor *TLR3* rs10025405 G allele was associated with reduced BrCa risk. The location of this SNP in the 3′ flanking region of the *TLR3* gene may alter mRNA expression or processing with resulting downstream effects on microbial nucleic acid recognition, intracellular signaling, and/or endosomal/lysosomal processing and degradation.

The *IRAK4* rs4251545 SNP codes for an Ala to Thr amino acid change at position 428. This non-synonymous mutation introduces a potential Ser/Thr kinase target and presents a bulkier and more polar residue. Further, the *IRAK4* rs4251545 locus occurs in an enhancer/silencer region of the gene, raising the additional possibility that this SNP may influence *IRAK4* transcription levels.

### Impact of *IRAK4* rs4251545 on subdomain conformation

In order to investigate the biochemical impact of the *IRAK4* rs4251545 locus on the aforementioned disease states, we first noted the location of the missense mutation coded by this SNP in the context of the IRAK4 protein. The most definitive structural data that includes amino acid 428 (coded by *IRAK4* rs4251545) is derived from x-ray diffraction of tetrameric crystals of the inhibitor-bound IRAK4 kinase domain (amino acid residues 160–460) (26). Figure 2 illustrates the IRAK4 kinase domain monomer in the context of the tetrameric crystal; amino acid 428 is represented in mauve, key residues of the activation loop are coded red, and key residues of the active binding site pocket are keyed green. We observed that amino acid 428 is located near the center of the penultimate αI helix on the surface of the truncated protein and faces the adjacent and almost parallel carboxy terminal α helix, αJ. We refer to the subdomain in which the *IRAK4* rs4251545 polymorphism resides as the extreme carboxy terminus (XCT). Specifically, the XCT, depicted in yellow within Figure 2, encompasses amino acid residues from approximately 418–460 and is comprised of the two carboxy terminal α helices (αI and αJ) and a putative 3/10 helix (with fewer residues per turn than the traditional α helix).

We performed MD simulations of the Ala428Thr variant to examine the impact of the *IRAK4* rs4251545 coded missense mutation on local XCT conformation at the surface of the IRAK4 kinase domain. Superposition of the wild-type Ala 428 and variant Thr 428 structures with respect to the backbone atoms of αI and the underlying αG and αE helices in dark blue, deep to αI and αJ, respectively, suggested differences in inter- and intra-helical dynamics between wild-type IRAK4 rs4251545 Ala and variant Thr alleles. Specifically, the Thr 428 variant enhances the H-bonding capabilities of αI and αJ, stabilizing them both. Replacing Thr for Ala also expands the inter-helical distance between αI and αJ, increasing the size of the groove between them.

## Discussion

Dysregulation of and genetic alterations in inflammation and immune response pathways have been linked with cancer susceptibility (6–9, 28). Although African-American women suffer disproportionately from more deadly forms of BrCa, only one study has explored the involvement of a single TLR-associated SNP (*TLR1/TLR6/TLR10* rs7696175) in relation to BrCa among this high risk sub-group (19). To explore whether other genetic susceptibilities in the TLR-pathway play a role in BrCa among African-American women, we examined 16 SNPs from 10 TLR-pathway genes in a pilot case-control study. We found that the *TLR3* rs10025405 and *IRAK4* rs4251545 loci under the additive genetic models were associated with significant alterations in BrCa risk among African-American women.

### TLR3

Inheritance of the homozygous minor *TLR3* rs10025405 G allele was associated with a fivefold reduction in BrCa risk. While the location of this SNP in the 3′ flanking region may alter mRNA expression/processing or gene product function (e.g., anti-tumor response, cell death), to our knowledge there are no published reports on the relationship between this *TLR3* SNP and human disease. However, there is direct and indirect pre-clinical as well as clinical evidence that TLR3 impacts breast tumor viability and/or behavior using TLR3-specific agonists, namely polyadenylic-polyuridylic acid [poly(A:U)] and polyinosinic-polycytidylic [poly(I:C)]. These agents are synthetic double-stranded RNA molecules that have been widely used as potent adjuvants in cancer immunotherapy, based on their ability to promote antigen-specific Th1-anti-tumor immune responses and boost antibody production when administered with antigen in mice (29–31). Salaun et al. (32) found that poly(A:U) treatment reduced breast tumor burden in immune compromised SCID/NOD mice, suggesting that the anti-tumor effects of poly(A:U) is attributed to TLR3 expressed by breast tumors rather than immune cells. The use of poly(A:U) increased BrCa patient survival and reduced tumor metastasis in two randomized clinical trials (33, 34). In addition, poly(A:U) specifically reduced metastatic relapse among patients who possessed TLR3 positive tumors in a recent European clinical trial (32). Consistent with this, treatment of three out of four human BrCa lines with poly(I:C) induced apoptosis *in vitro* (35), and poly(A:U) inhibited HCC1806 BrCa tumor growth *in vitro* and *in vivo* (32).

Given that tumor-expressed TLR3 appears to promote apoptosis in transformed breast epithelia, it is reasonable to speculate that the *TLR3* rs10025405 variant (protective among the African-American women in our pilot study) alters TLR3 expression. In addition, the mechanism of TLR3-triggered BrCa apoptosis also requires downstream signaling via TRIF/TRAM1 (MyD88-independent) and IRAK4 in Cama-1 human BrCa cells (35–37). Interestingly, in these cells type I IFN enhanced but could not initiate apoptosis (35). Thus, even if the *TLR3* rs10025405 minor variant alters TLR3 expression, other factors, such as those that influence TLR3 regulation, localization, and/or signaling pathways, are also likely to impact TLR3-triggered BrCa apoptosis.

### IRAK4

This report also finds that the *IRAK4* rs4251545 variant in the XCT subdomain of IRAK4 (amino acids 418–460) is associated with a fivefold increase in BrCa risk among African-American women. While the minor variant of this SNP has been associated with Gram-positive bacterial infections (38), this report is the first to correlate this or any IRAK4 polymorphism with BrCa risk. *IRAK4* rs4251545 simultaneously impacts a putative enhancer/silencer site (and subsequent IRAK4 expression levels) and alters the amino acid sequence (and possibly IRAK4 structure, phosphorylation state, kinase activity, and/or protein–protein interactions). We can envision a variety of scenarios in which the *IRAK4* rs4251545 minor variant might promote BrCa risk by altering the expression or phosphorylation state of IRAK4 or its interaction with upstream adaptors (like MyD88) and/or downstream kinases (like IRAK1 and IRAK2) and E3 ubiquitin ligases (like TRAF6). Given our data on *TLR3*, it is also possible *IRAK4* rs4251545 impairs TLR3-mediated apoptotic signaling and aids the survival of transformed breast epithelia. IRAK4 was first observed in the serum of both normal individuals and those with renal carcinoma (39). Further, host peptides corresponding to the IRAK4 XCT (aa 417-441) were isolated from the serum of MCF7 BrCa xenografted mice (40). These findings prompt intriguing questions about the biological impact of IRAK4 trafficking and processing and the role of the XCT fragment.

The association of IRAK4 with BrCa risk in African-American women is not unexpected. Given that IRAK4 is a common mediator of TLR signaling from the cell surface and from endosomes (Figure 1), the fact that all TLRs except TLR8 have been shown to influence BrCa progression (9) implicates IRAK4 as an accomplice. Figure 1 illustrates representative signaling pathways from surface (TLR2 and TLR4) and endosomal (TLR3 and TLR4) TLRs [reviewed in Ref. (41)] that converge at IRAK4. It is well-known that IRAK4 is activated by upstream signaling adaptor MyD88 docking via their shared death domains and complexes with IRAK1 to activate downstream E3 ubiquitin ligase TRAF6. However, it should be noted that TLR-pathway linked IRAK4 responds to both MyD88-dependent and -independent upstream signals and prompts both TRAF6-dependent and -independent downstream signaling (35, 42). As shown in Figure 1, IRAK4 mediated signaling results in a wide range of cancer-related cellular responses, including: imbalances in cell survival/cell death; production of pro-inflammatory cytokines and chemokines; expression of interferons and interferon-inducible genes; and release of matrix metalloproteinases that degrade extracellular matrix and facilitate cell migration/metastasis.

### Strengths, limitations and future directions

This report is only the second of its kind to describe the impact of innate immunity sequence variants on BrCa risk among African-American women. Under the additive genetic model, we observed significant alterations in the risk estimates among carriers of the *TLR3* rs10025405 and *IRAK4* rs4251545 variant alleles, despite the use of individuals with benign disease as controls. However, if disease-free individuals had served as controls, the observed risk estimates for the *TLR3* and *IRAK4* loci would have been even more extreme, since disease-free individuals would be expected to have a smaller tendency to develop BrCa than individuals with benign nodules. In other words, the difference in risk between benign controls and BrCa patients is smaller than the difference in risk between disease-free controls and BrCa patients. While the risk estimates were not adjusted for population admixture, which is a common issue among African-Americans, several genetic epidemiology studies indicate that adjustment for population stratification only results in a ±0.02 change in the risk estimates relative to unadjusted models (28, 43–47). Despite the large differences in age between cases and controls, we did not observe significant variations in the risk estimates comparing age-adjusted and unadjusted risk estimates.

A variety of factors may confound the relationship between variant innate immunity markers and BrCa. In order for a factor to serve as a confounder, it must be related to BrCa risk and the expression or function of the innate immunity markers. It is common knowledge that the risk of developing BrCa increases with long-term exposure to estrogen due to late age at menopause, late age at first birth, null parity, overweight/obesity, use of oral contraceptives, and use of hormone replacement therapy. In addition, there are limited published reports that suggest exposure to estrogen may alter the production of inflammatory mediators and cytokines via activation of TLR3 and TLR4 (48, 49). Consequently, the aforementioned reproductive/weight risk factors may in fact alter the inflammatory response activities of TLR3 and IRAK4. Unfortunately, we failed to adjust our risk estimates for these two factors and did not acquire this information from study participants. However, we have considered how our risk estimates may have varied if they had been adjusted for the aforementioned potential confounders. Following adjustment for surrogates of long-term estrogen exposure, we would predict an increase in BrCa risk estimates for the *TLR3* and *IRAK4* loci when compared to unadjusted models, based on the observation that long-term estrogen exposure enhances the production of inflammatory mediators in response to TLR4 (and downstream IRAK4) activation. Specifically, chronic estrogen exposure promotes TLR4-induced pro-inflammatory mediator production (IL-1-β, IL6, TNF-α) in macrophages *in vivo* (48). In contrast, short-term estrogen exposure elicits an anti-inflammatory response via TLR3, suggesting a corresponding decrease in BrCa risk (49). Interestingly, short-term exposure to estrogen (i.e., 17 β-estradiol) does not influence TLR3 mRNA/protein expression in endometrial cell lines, but modulates TLR3 function by suppressing TLR3-ligand induced cytokine/chemokine production (49). Clearly, additional studies are needed to address the important question of whether long-term estrogen exposure influences TLR3 or IRAK4 expression or function in *in vitro* and *in vivo* BrCa models.

Due to our limited sample size, we did not have ample statistical power to assess whether the risk of developing BrCa would vary according to disease severity (i.e., high tumor stage, tumor size ≥2 cm, invasive pathology, and triple negative BrCa). In an exploratory analysis of the current study, innate immunity sequence variants [IRAK2 (rs242724, rs6442161), IRAK4 rs4251545), OAS1 rs10774671, CD14 rs2569188, and TLR4 (rs913930, rs7045953)] were significantly related to disease aggressiveness. For instance, IRAK4 rs4251545 was associated with a 2.56-fold increase in invasive disease (OR = 2.56; 95% CI = 1.1–5.91); whereas, inheritance of the IRAK2 rs242724 minor alleles were linked to non-aggressive disease (i.e., tumor size <2 cm, ER + /PR + /Her-2 neu+ status).

From a statistical point of view, our results can only be considered suggestive and will require additional evaluation and validation in larger multi-center studies. Nevertheless, the potential biological and clinical implications are compelling. In this context, future studies will include sequencing of innate immunity genes, including *TLR3* and *IRAK4*, as well as genes that directly or indirectly interact with these two markers. Such an approach will allow us to identify, evaluate and validate biomarkers of innate immunity that may ultimately serve as potential diagnostic, prognostic, and/or clinical management tools. Such efforts are likely to provide further understanding of the consequences of altered innate immunity on BrCa tumor progression. Functional studies are also needed to determine whether genetic changes in the putative enhancer/silencer site of the IRAK4 gene and/or structural changes in IRAK4 due to the Ala248Thr missense mutation alter the stability, activity, and/or protein–protein interactions of this regulatory kinase. Future studies will also address downstream effects of TLR3 and IRAK4 alterations on immune/inflammatory responses and tumor behavior using *in vitro* and *in vivo* models. Currently, our laboratory is developing and exploring the effects of strategically designed IRAK4 agonists and antagonists on the behavior of transformed breast epithelia.

## Conclusion

Our findings suggest that two mediators of innate immunity signaling, TLR3 and IRAK4, may impact BrCa development among African-American women. However, our results are preliminary and require further evaluation in larger, ethnically diverse populations.

## Conflict of Interest Statement

The authors declare that the research was conducted in the absence of any commercial or financial relationships that could be construed as a potential conflict of interest.

## Footnotes

^1^http://snpinfo.niehs.nih.gov/snpinfo/snpfunc.htm

^2^www.ncbi.nlm.nih.gov

^3^http://www.autoprimer.com

## Abbreviations

BrCa, breast cancer; CHARMM22, Chemistry at HARvard Macromolecular Mechanics 22; CI, confidence interval; *IRAK4*, interleukin-1 receptor-associated kinase 4 gene; IRAK4, interleukin-1 receptor-associated kinase 4 protein; NAMD, not (just) another molecular dynamics simulation software; OR, odds ratio; SNP, single nucleotide polymorphism; TIP3, 3-site rigid water model; TLR, toll-like receptor; XCT, extreme carboxy terminus.
